# The critical role of glucose deprivation in epithelial-mesenchymal transition in hepatocellular carcinoma under hypoxia

**DOI:** 10.1038/s41598-020-58124-1

**Published:** 2020-01-30

**Authors:** Hanhee Jo, Jongsook Lee, Jeongyong Jeon, Seon yoo Kim, Jee-in Chung, Hae yong Ko, Misu Lee, Mijin Yun

**Affiliations:** 1 Division of Life Sciences, College of Life Science and Bioengineering, Incheon National University, Incheon, South Korea; 2 Department of Nuclear Medicine, Severance Hospital, Yonsei University College of Medicine, Seoul, South Korea

**Keywords:** Cancer microenvironment, Cancer microenvironment, Metastasis, Metastasis

## Abstract

Imaging with 18F-fluorodeoxyglucose (FDG) positron emission tomography/computed tomography (PET/CT) is used to determine sites of abnormal glucose metabolism to predict high tumor grade, metastasis, and poor patient survival. However, not all tumors with increased 18F-FDG uptake show aggressive tumor biology, as evident from the moderate correlation between metastasis and high FDG uptake. We hypothesized that metastasis is likely attributable to the complexity and heterogeneity of the cancer microenvironment. To identify the cancer microenvironment that induces the epithelial-mesenchymal transition (EMT) process, tumor areas of patients with HCC were analyzed by immunostaining. Our data demonstrated the induction of EMT process in HCC cells with low proliferation under hypoxic conditions. To validate our finding, among HCC cell lines, HepG2 cells with highly increased expression of HIF1α under hypoxia were employed *in vitro* and *in vivo*. Major changes in EMT-associated protein expression, such as the up-regulation of N-cadherin and snail/slug are associated with decreased proliferation-related protein (PCNA) caused by glucose deprivation under hypoxia. Indeed, PCNA knockdown-HepG2 cells under hypoxia showed the induction of more EMT process compare to the control. Thus, HCC cells with low proliferative potential under glucose-deprived and hypoxic conditions show high probability for induced EMT process and promote cell invasion. This study investigates reasons as to why an EMT process cannot fully be predicted. Our observations indicate that rather than analyzing a single factor, an integrated analysis of hypoxia with low glucose metabolism and low cell proliferation might be helpful to predict the potential impact on induction of EMT process and promotion of cell invasion.

## Introduction

Hepatocellular carcinoma (HCC) is one of the leading causes of cancer-related deaths worldwide and is associated with various risk factors such as hepatitis virus infection, aflatoxin exposure, fatty liver, or alcohol abuse^1,2^. HCC is a clinical, metabolic, and heterogeneous tumor with various phenotypes^1–6^. Tumors include proliferating, slow dividing, quiescent or necrotic/apoptotic tumor cell populations, as well as fast- or slow-migrating tumor cells^7,8^. Genetic heterogeneity in solitary disseminated tumor cells and metastasis has been shown by genetic and expression profiling studies^9–15^. One of the known causes of heterogeneity related to rapid cellular proliferation is the formation of abnormal vascular networks characterized by leaking and compressed blood and lymphatic vessels, creating hypoxic areas in the tumors. Tumor hypoxia induces metabolic reprogramming from mitochondrial oxidation to glycolysis and drug resistance by activating pathways controlled by hypoxia-inducible factor (HIF)^16,17^. Moreover, there is a close relationship between hypoxia and tumor metastasis that leads to poor prognosis^18–20^.

Epithelial-mesenchymal transition (EMT) is a complex trans-differentiation process that increases the migratory and invasive ability of tumor cells and is an early step in cancer metastasis^21,22^. EMT process is associated with the proliferation capacity of cancer cells. It is known that mesenchymal-like tumor cells gain movement ability at the expense of proliferative potential by cell cycle arrest^23–25^. It involves complex cell reprogramming that renders the cells resistant to anti-cancer drugs for primary tumors and promotes cancer stemness properties^26,27^. Members of the classical cadherin family, such as N-cadherin and Vimentin, have been proposed as inducers of the EMT process^28^. The repression of E-cadherin during EMT is mediated by the binding of EMT transcription factors, including Snail and Slug^26^. The heterogeneity of the EMT spectrum has an impact on metastasis, resulting in some cells of the primary tumor colonizing the secondary site^29,30^. It remains controversial whether tumor cells innately have the ability to metastasize, or whether the metastatic ability is gained from a set of genetic and/or other changes that occur throughout tumor progression or from the tumor microenvironment.

Positron emission tomography/computed tomography (PET/CT) imaging for F-18 fluorodeoxyglucose (18F-FDG), a surrogate marker for measuring enhanced glycolysis, has been useful in capturing hypoxia-related metabolic reprogramming in patients with HCC. So far, HCC with increased FDG uptake on PET/CT imaging is associated with a higher frequency of metastasis^31,32^. However, as observed in a previous study, more than 50% of HCC patients with increased FDG uptake do not display tumor metastasis^33^. We hypothesized in this study that the remaining HCC patients with high FDG uptake may fail to promote metastasis because of the insufficient tumor microenvironment to induce the EMT process. The current study, therefore, aimed to further investigate the synergistic effects of glucose availability, hypoxia, and cellular proliferation on EMT *in vitro* using HCC cell lines and *in vivo* using xenografts and human HCCs.

## Results

### Hypoxia, GLUT1, Ki67, and EMT in human HCC specimen according to FDG uptake upon PET/CT

**Figure 1:**
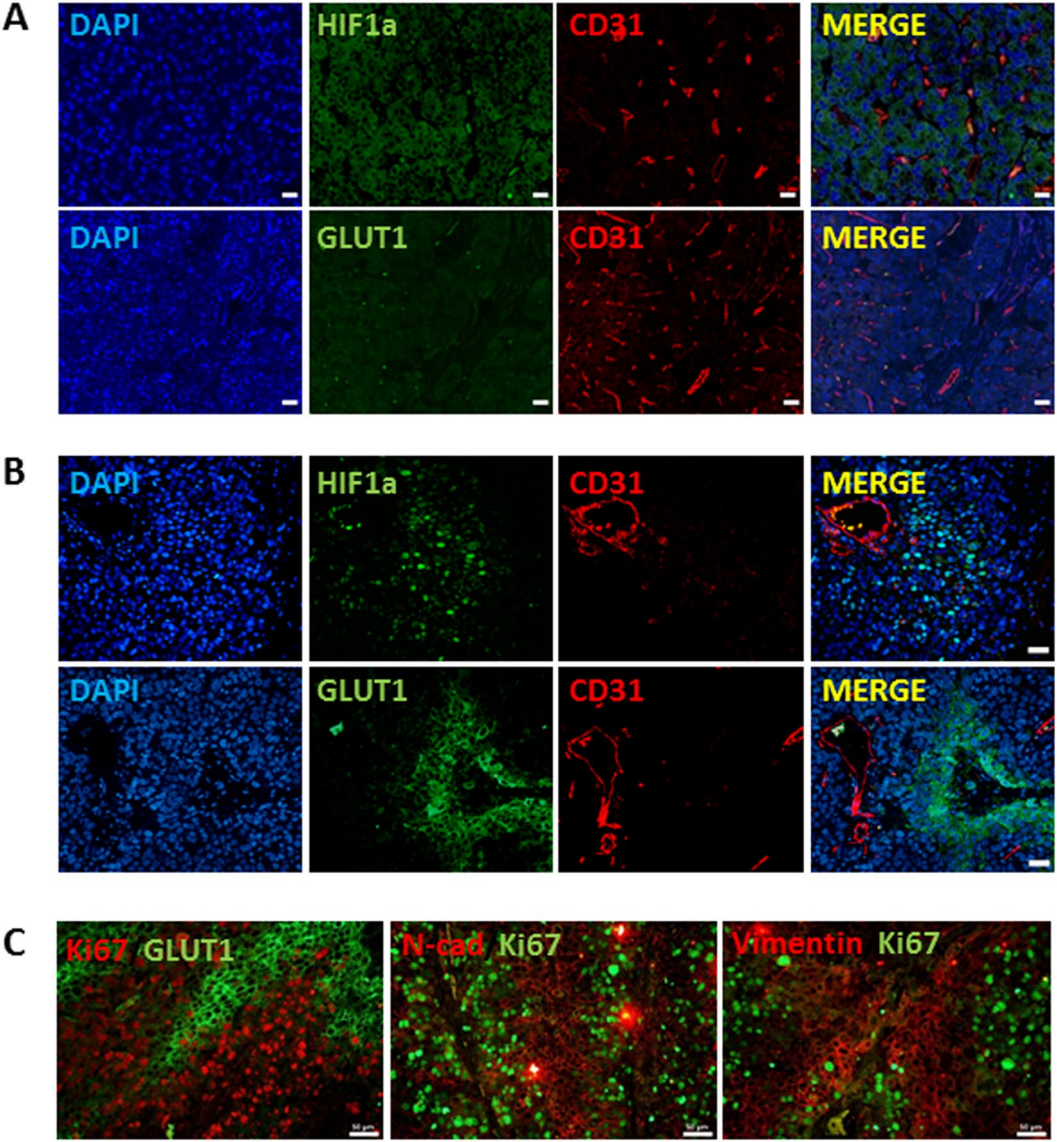
Differences in the expression of HIF1α, GLUT1, CD31, and EMT-related proteins in HCC samples based on 18F-FDG uptake. (**A**,**B**) Expression of HIF1α, GLUT1, and CD31 in human HCC with (**A**) low 18F-FDG uptake or (**B**) high 18F-FDG uptake. Immunostaining with antibodies against indicated proteins performed on FFPE tissues. Nuclei were counterstained with DAPI. (**C**) Immunofluorescence detection using antibodies against GLUT1, vimentin, and Ki67 in FFPE tissues from human patients with HCC. Nuclei were counterstained with DAPI. Scale bars: 100 μm.

As described in the Methods section, patients with HCC were divided into two groups depending on glucose uptake – 10 patients with HCC showed high 18F-FDG uptake (TLR > 1.62) and 13 showed low 18F-FDG uptake (TLR≤1.62) on PET/CT imaging. Expression of HIF1α, GLUT1, and CD31 was investigated in these two groups. Tumor region of HCC tissue from HCC patients with high FDG uptake showed statistically significantly more positive regions of Hif1α expression and membranous GLUT1 as compared with HCC with low FDG uptake by immunostaining (Fig. 1A, Supplementary Fig. S2A,B). In HCC patients with high FDG uptake, there was no change in CD31 expression, but rather an uneven distribution of blood vessels in the tumor region (Fig. 1A and Supplementary Fig. S2C). In contrast, HCCs with low 18F-FDG uptake showed decreased expression of HIF1α and GLUT1, and well-organized, uniformly branched blood vessels, as confirmed by CD31 staining (Fig. 1B and Supplementary Fig. S2C). Next, qRT-PCR was carried out to determine the expression of the EMT-related genes, *HIF1α* and proliferating cell nuclear antigen (*PCNA)*, as proliferation markers. Based on the qRT-PCR result, we observed statically no correlation between *HIF1α* and *SNAl1* (R^2^ score = 0.04), *HIF1α* and *CDH2* (R^2^ score = 0.21), *PCNA* and *SNAl1* (R^2^ score = 0.1), and *PCNA* and *CDH2* (R^2^ score = 0.023) (Supplementary Fig. S4A). In addition, a TCGA analysis was performed for 360 human HCC samples (Supporting Fig. 4B). Spearman’s correlation analysis showed a significant positive correlation between EMT-related genes and the *HIf1α* gene. Interestingly, there is a significant, but weak negative correlation between the expression levels of *SNAIL1* and *PCNA* mRNAs (Spearman r =−0.1885, P = 0.0003). To confirm mRNA expression data, GLUT1, Ki67, and EMT- related proteins were immunostained in hypoxia positive regions of human HCC. Most of the hypoxic areas showed expression of GLUT1, but not Ki67. Moreover, there were negative correlations between Ki67 expression and that of the EMT-related proteins, N-cadherin or vimentin (Fig. 1C and Supplementary Fig. S2D). Altogether, our data demonstrate that EMT-related proteins, such as N-cadherin or vimentin are differentially expressed in HCC cancer cells depending on the proliferative rate under hypoxic conditions.

### Sensitivity of different HCC cell types to hypoxic conditions

**Figure 2:**
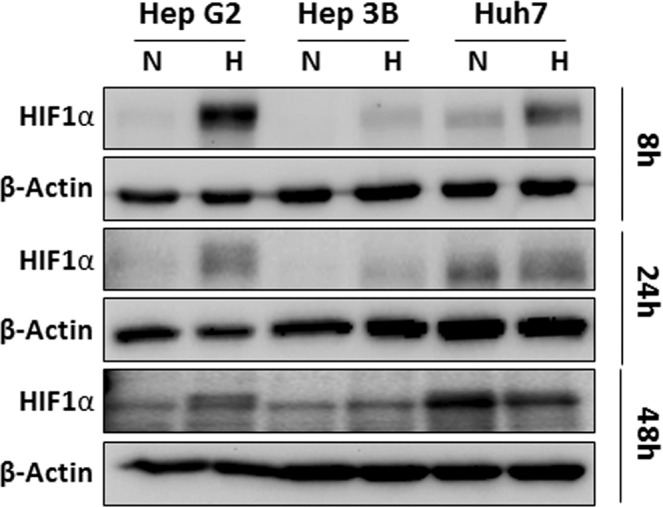
Response of HCC tumor cells to hypoxia. (**A**) Expression of HIF1α under hypoxic conditions. Three HCC cell lines (HepG2, Hep3B, and Huh7) were exposed to hypoxic conditions (1% oxygen). After the indicated incubation period, HIF1α and β-actin expression levels were evaluated by western blotting. Data shown represents one of three independent experiments with similar results.

Our previous study showed that different types of HCC cells have different characteristics, including the uptake of glucose^34^. To confirm the results obtained from patients with HCC, we decided to select the HCC model system that mimics the heterozygous hypoxic conditions of patients with HCC. We examined HIF1α expression under hypoxic and normoxic conditions in three HCC cell lines (HepG2, Hep3B, and Huh7). HepG2 and Hep3B cells showed increased expression of HIF1α after 8 and 24 h of exposure to hypoxic conditions (Fig. 2A). However, Huh7 cells, which have a high expression of HIF1a under normoxia, showed a mild increase in the expression of HIF1a under hypoxic conditions. HepG2 and Hep3B xenograft models showed varying expression levels of GLUT1 and Ki67 depending on heterogeneous CAIX expression, a hypoxia-inducible metal-enzyme that promotes cancer cell survival and invasion via HIF1α activation (Supplementary Fig. S3). Therefore, HepG2 and Hep3B cells were selected to investigate the biologic effects of hypoxic conditions in HCC for further *in vitro* studies. In contrast, the Huh7 model has a high homogenous expression of CAIX, GLUT1, and Ki67 in most tumor regions.

### Dysregulation of proliferation and EMT under oxygen deprivation in hypoxia-sensitive HepG2 cells

**Figure 3:**
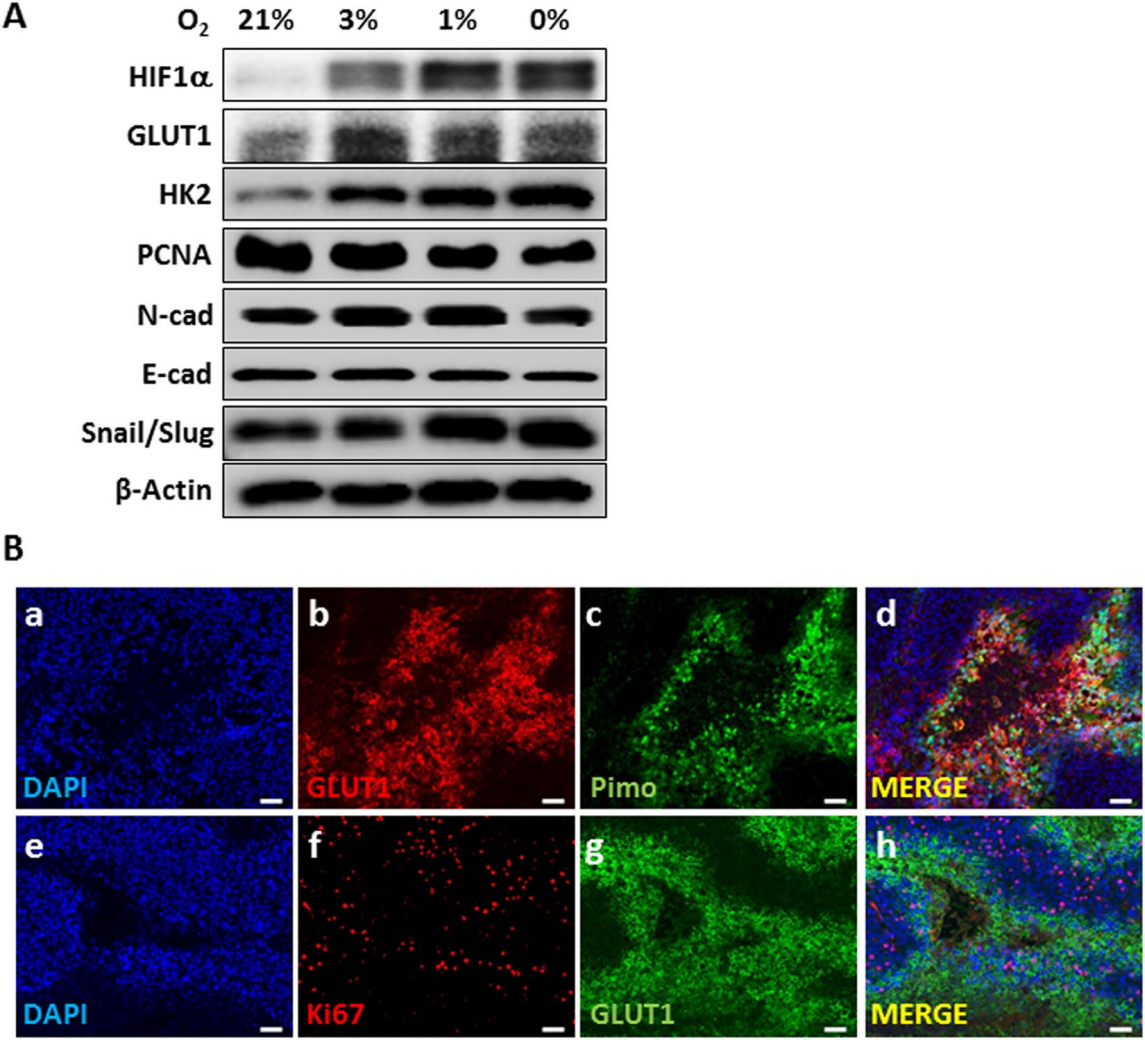
Expression of hypoxia-related proteins in HepG2 cells. (**A**) HepG2 cells were exposed to the indicated hypoxic conditions. After 24 h, HIF1α, GLUT1, HK2, PCNA, N-cadherin, E-cadherin, Snail/Slug, and β-actin expression levels were evaluated by western blotting. Data shown represents one of three independent experiments with similar results. (**B**) Immunofluorescent staining with antibodies against (b, g) GLUT1, (c) pimonidazole, and (f) Ki67 performed on FFPE tissues from HepG2-xenograft model. Nuclei were counterstained with DAPI. In parallel, immunohistochemistry was performed using HIF1α antibodies. Scale bars: 100 μm.

To investigate the oxygen concentration-dependent changes, HepG2 cells were cultured for 24 h under 0%, 1%, 3%, and 21% oxygen concentration. The expression of HIF1α was upregulated under hypoxia (3% and below) (Fig. 3A). The expression of PCNA, an indicator of proliferation, was reduced at 1% and 0% oxygen. Unlike PCNA, the glycolysis-related proteins, HK2 and GLUT1, showed continuously increased expression according to the oxygen concentration (Fig. 3A). Furthermore, the expression of the regulatory transcriptional factors of EMT, Snail/Slug and N-cadherin, was determined. Under severe hypoxic condition (below 1% oxygen), Snail/Slug was upregulated. The expression of N-cadherin was increased under mild hypoxia (3% and 1% oxygen). Thus, we determined the effect of hypoxia on glucose metabolism and proliferation-related protein *in vitro*.

### Hypoxia versus Ki67, GLUT1, and EMT expression in HepG2-xenograft model

**Figure 4:**
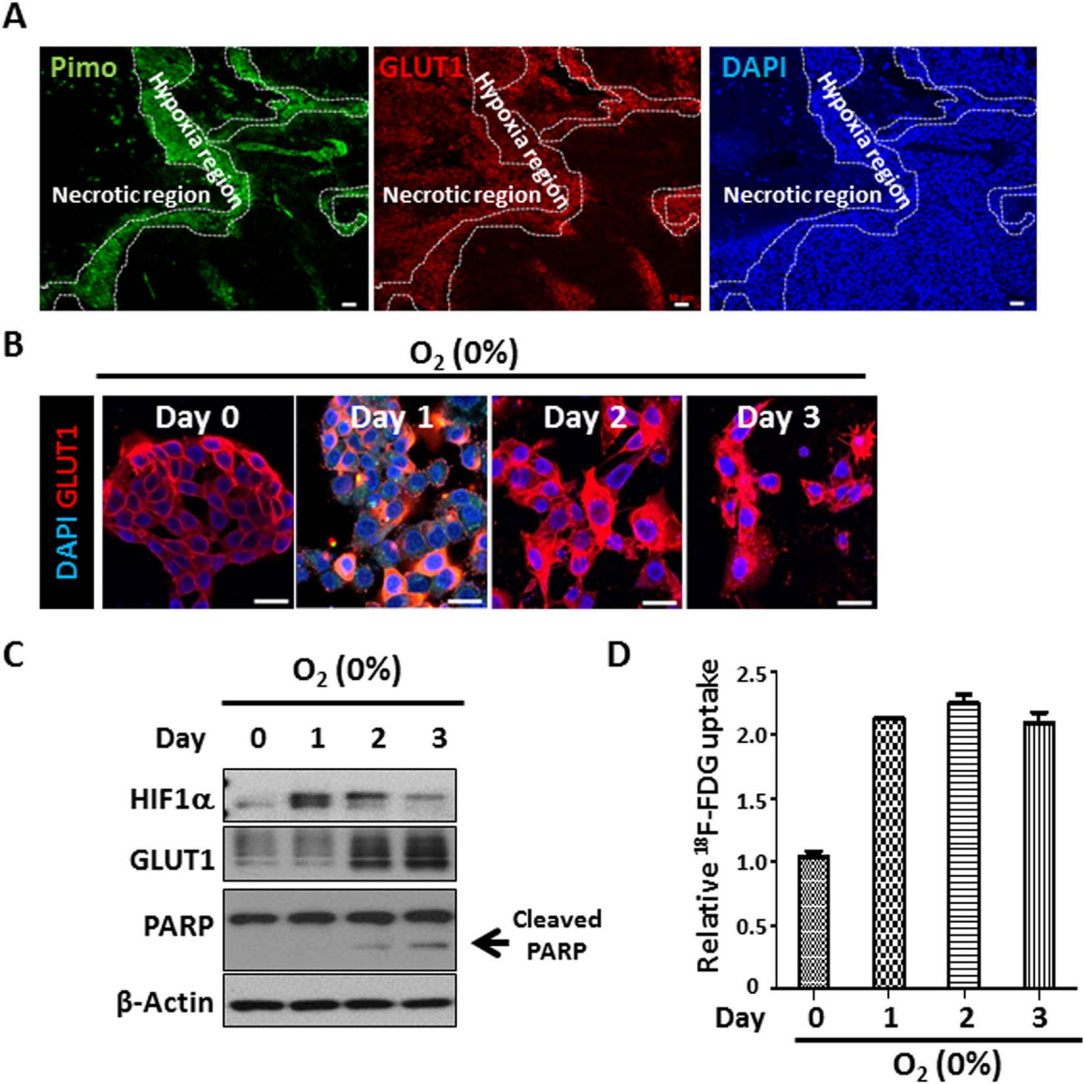
GLUT1 expression in the necrotic region. (**A**) Expression of pimonidazole and GLUT1 in hypoxia and necrotic region of HepG2-xenograft model. Immunostaining with antibodies against indicated proteins performed on FFPE tissues from HepG2-xenograft model. Nuclei were counterstained with DAPI. (**B**) HepG2 cells were plated on coverslips in 24-well plates and incubated under severe hypoxic condition (0%). After indicated incubation times, the cells were fixed and immune-stained for GLUT1 protein. (**C**) HIF1α, GLUT1, and actin expression levels were measured using western blotting. Data shown represents one of three independent experiments with similar results. (**D**) In samples parallel to “C,” the uptake of 18F-FDG was measured using a gamma-counter (right panel). The data were analyzed independently using three replicates each (n = 3). Data are presented as means ± standard deviations.

In the HepG2-xenograft model, the expression of GLUT1 (b), the distribution of pimonidazole staining (c, as a hypoxia marker), and the expression of Ki67 (f) were assessed in various tumor regions to determine the effect of hypoxia on glucose metabolism and cellular proliferation (Fig. 3B). Hypoxic regions with positive pimonidazole staining showed decreased Ki67 expression compared with that in non-hypoxia region with negative pimonidazole staining. These results were consistent with those obtained for areas with hypoxia (≤3% oxygen) and decreased cellular proliferation. In HepG2-xenograft model, we observed the upregulation of GLUT1 expression in the pimonidazole-positive hypoxic regions, and it was continuously expressed in some of the necrotic areas, regardless of pimonidazole staining (Fig. 4A). Rapidly growing tumors suffer from oxygen deprivation for an extended period due to insufficient blood supply, resulting in necrotic tumor cell death in the core region of tumors. Therefore, we cultured HepG2 cells under oxygen-free environment for 3 days under 0% oxygen. The expression of HIF1α was increased after one day under oxygen deprivation. However, following increased incubation time with 0% oxygen, the expression of HIF1α was reduced and that of GLUT1 was increased. Thus, even if our experimental conditions could not mimic the necrotic region, we assumed that a 3-day incubation with 0% oxygen could mimic the necrotic region. Thus, our result indicates that GLUT1 expression was positive in the necrotic region because the few cells that survived under oxygen deprivation for an extended period still maintained GLUT1 expression (Fig. 4B,C). We also measured the uptake of radiolabelled glucose in HepG2 cells by *in vitro* binding assays to determine whether GLUT1 still functions in the necrotic area. The uptake of radioactive glucose persisted for 3 days, even under oxygen deprivation (Fig. 4D). Thus, GLUT1 expression may not be a sufficient marker of inducing EMT process.

### Synergistic expression of EMT-related proteins under hypoxia and glucose deprivation

**Figure 5:**
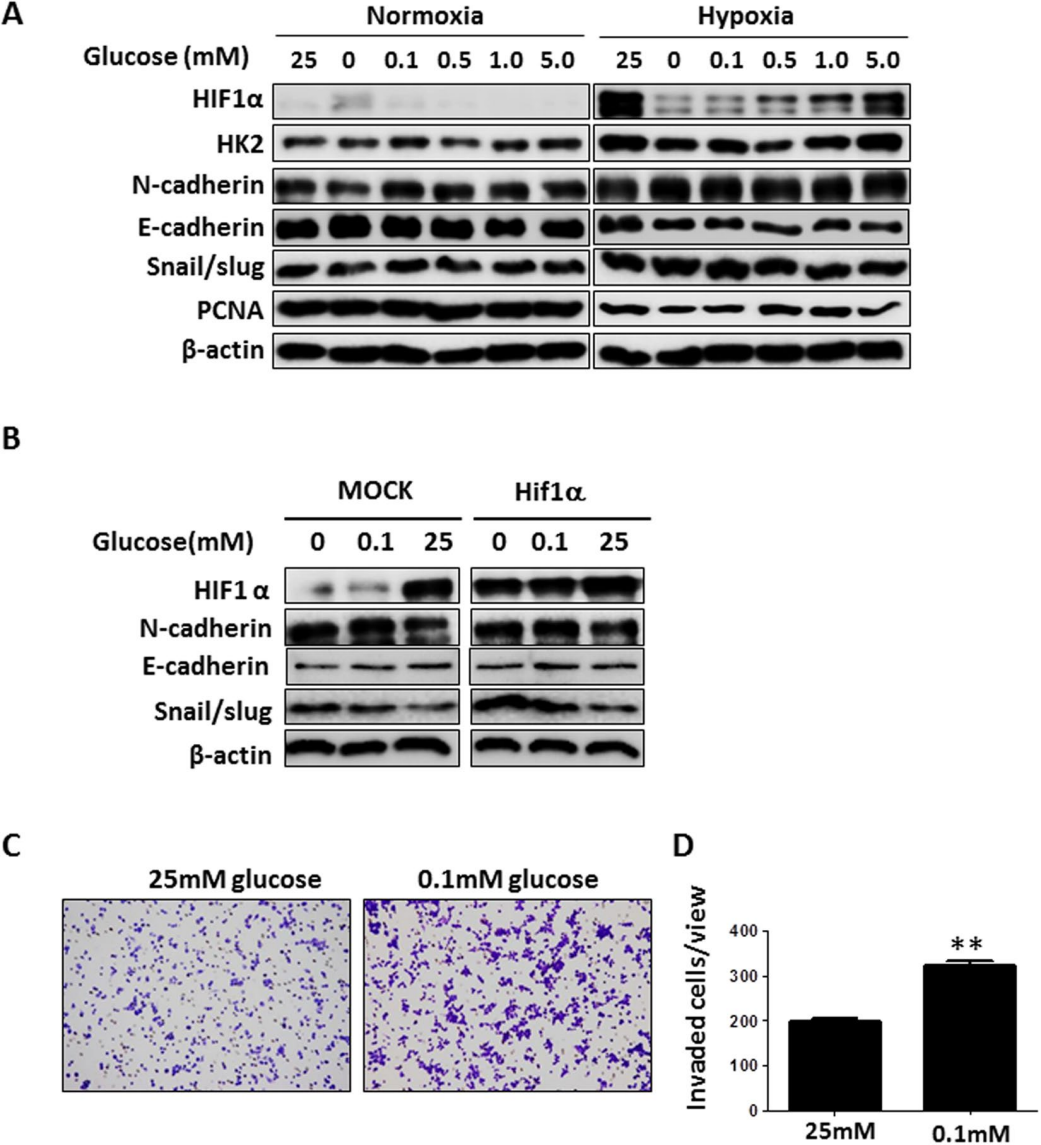
Increased EMT-related protein expression after glucose-deprivation under hypoxia. (**A**) HepG2 cells were incubated with various concentrations of glucose under normoxia or hypoxia (1% oxygen). After 8 h, HIF1α, GLUT1, HK2, N-cadherin, E-cadherin, Snail/Slug, PCNA, and β-actin expression levels were examined by western blotting. The images have been cropped from those of different blots exposed for the same time. Data shown represents one of three independent experiments with similar results. (**B**) HepG2 cells were transfected with MOCK or pCMV3- HIF1α. After 24 h, HepG2 cells were incubated with the indicated concentration of glucose under hypoxia (1% oxygen) for 8 h. HIF1α, N-cadherin, E-cadherin, Snail/Slug, and β-actin expression levels were then examined by western blotting. The images have been cropped from those of different blots exposed for the same time. Data shown represents one of three independent experiments with similar results. (**C**) HepG2 cells were incubated with the indicated concentration of glucose under hypoxia (1% oxygen) for 8 h. HepG2 cells penetrating the membrane were fixed and visualized by crystal violet staining. (**D**) Quantitative analyses were performed for the cells migrating through the Matrigel-coated filter. Five random fields of each test at ×200 magnification were counted (±standard deviation) in 3 independent experiments. *p < 0.05.

In the core of tumors, there might be extreme metabolic stress resulting from oxygen and glucose depletion. Unlike the hypoxia region stained by pimonidazole, the actual availability of glucose in the diffusion restricted regions showing increased GLUT1 expression could not be measured in the xenograft. Therefore, the synergistic effect of glucose and oxygen deprivation under hypoxia on the expression of EMT-related proteins was assessed *in vitro*. As expected, cell proliferation was reduced after glucose deprivation under 1% hypoxic condition (Supplementary Fig. S5). However, as shown in Fig. 5A, severe glucose deprivation at 1% oxygen concentration increased the expression of N-cadherin and Snail/Slug, suggesting that glucose deprivation and hypoxia synergistically increased EMT-related proteins. Indeed, glucose deficiency-mediated dysregulated expression of EMT-related proteins was less than that in the hypoxic state under normoxic conditions. Interestingly, although the expression of EMT-related proteins was upregulated, HiF1α expression was abolished in HepG2 cells grown in a glucose-deprived medium under hypoxia (Fig. 5A). We observed similar results using Hep3B cells (Supplementary Fig. S6A). To further validate our findings, Hif1α was overexpressed in HepG2 cells and Hep3B cells exposed to different concentrations of glucose (0, 0.1 and 25 mM) under hypoxia. The increased Hif1α expression also reduced after incubation of cells in a low or no glucose medium under hypoxia (Fig. 5B and Supplementary Fig. S6B). The expression of EMT-related proteins was downregulated with the increase in the concentration of glucose in both MOCK- and HIF1α-transfected HepG2 cells. HepG2 cells (Fig. 5C and D) and Hep3B cells (Supplementary Fig. S6C,D) incubated with 0.1 mM glucose under hypoxia exhibited a higher invasion potential than did those incubated with 25 mM glucose. Usually, it is difficult to identify EMT-positive tumor cells in a hypoxia-negative region *in vivo* and in patients with HCC. Thus, we treated HepG2 cells with glucose deprivation medium under hypoxia with glutamine, another major nutrient in cancer metabolism. The glucose deprivation-induced reduction of Hif1α recovered after treatment with glutamine (Supplementary Fig. S7A). Moreover, the expression of Hif1α in the glutamine transporter ASCT2-positive region was much more than that in the ASCT2-negative region in the HepG2-xenograft model (Supplementary Fig. S7B). Taken together, these results suggest that increased EMT was associated with glucose deprivation under hypoxia in HCC cells.

### Synergistic expression of EMT-related proteins under hypoxia and proliferation

**Figure 6:**
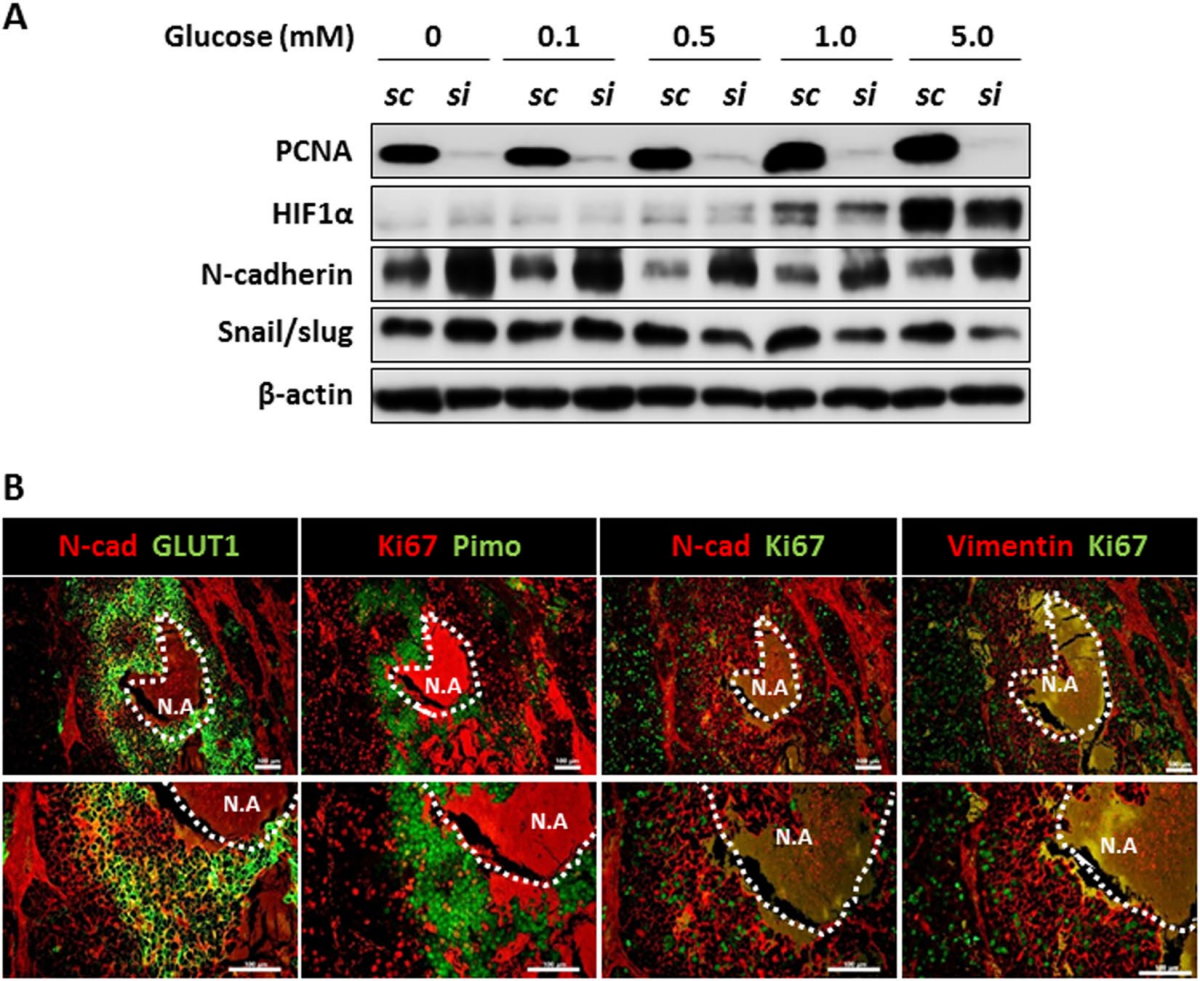
Increased EMT-related protein expression in low proliferative cells under hypoxia. (**A**) HepG2 cells were transfected with scrambled siRNA oligos or siRNA oligos against PCNA. After 24 h, HepG2 cells were incubated with various concentrations of glucose under hypoxic (1% oxygen) condition for 8 h. PCNA, HIF1α, N-cadherin, Snail/Slug, and β-actin expression levels were then examined by western blotting. Data shown represents one of three independent experiments with similar results. (**B**) Immunofluorescent staining with indicated antibodies performed on FFPE tissues from HepG2-xenograft model. Nuclei were counterstained with DAPI. Scale bars: 100 μm. N.A; necrotic area.

In our study, we mimicked the microenvironment that induces the EMT process. In Fig. 5, we showed that, under hypoxic conditions, glucose-deprived HCC cells upregulated the expression of N-cadherin and snail/slug and downregulated E-cadherin. Interestingly, HCC cells with 0 mM and 0.1 mM glucose showed a decrease in PCNA as compared to HCC cells with high glucose (Fig. 5A). Thus, our hypothesis is that the induction of EMT-related proteins partly depends on the proliferation rate of HCC cells. Proliferating cell nuclear antigen (PCNA) is known as a molecular marker for proliferation^35^. Thus, PCNA expression was knocked down in HepG2 HCC cells, which were then incubated with various glucose concentrations under hypoxic conditions. Knockdown of PCNA in HepG2 and Hep3B cells significantly increased the expression of N-cadherin and Snail/Slug in the complete absence of glucose under hypoxia (Fig. 6A and Supplementary Fig. S8A). However, the addition of glucose to the medium reduced the induction of EMT-related proteins in PCNA-KD HCC cells. These results showed that the proliferation of HCC cells also plays an important role in induction of EMT process.

Next, we determined whether the expression of EMT-related proteins in hypoxic regions changes depending on the oxygen concentration *in vivo*. Necrotic regions are commonly found in the core region in response to extreme metabolic stress resulting from oxygen and glucose depletion. Because we could not measure glucose deprivation directly in the tumor tissue, we examined the co-expression of EMT-proteins with Ki67 next to the necrotic region. As shown in Fig. 6B, the expression of Ki67 varied depending on the distance from the necrotic region. Similar to the *in vitro* experiments, we observed a negative correlation between Ki67 and expression of N-cadherin or vimentin. Thus, glucose deprivation under a hypoxic condition effectively dysregulates EMT-related proteins *in vivo*. Thus, our data reveal a unique microenvironment comprising glucose deprivation, hypoxia, and low proliferation rate for inducing EMT process under hypoxia.

## Discussion

The main aim of the current study was to investigate the microenvironment factors that are favorable for metastasis in HCC. Previous studies suggested that high expression of HIF1α and GLUT1 in HCC is a major causative factor for metastasis^36,37^. However, our current data suggest that the EMT process is very complex and is caused by a combination of hypoxia, low cell proliferation, and poor glucose diffusion.

Previous studies suggested that high expression of HIF1α^38–40^ and GLUT1^41,42^ in HCC are major causative factors for metastasis. The rapid growth rate of cancer cells induces blood vessel leakage, chaotic structure, and non-laminar blood flow. The blood vessels formed in the tumor do not undergo normal physiological angiogenesis, and the nutritional supply to the tumor is abnormal. Accordingly, the core of the tumor might have extreme metabolic stress such as not only oxygen but also nutrient depletion. Therefore, hypoxia itself may not be sufficient to mimic the microenvironment of the tumor. In this study, the synergistic effect of glucose and oxygen deprivation under hypoxia on the expression of EMT-related proteins was assessed *in vitro*. We found that severe glucose deprivation in addition to hypoxia played an additive role in increasing the expression of N-cadherin as well as Snail/Slug. It seems that glucose deprivation and hypoxia acted synergistically to increase EMT-related proteins. In addition, our study demonstrated a reduction in endo- and exogenous HIF1a expression by glucose deprivation under hypoxia. Similar to our study, the study conducted by Kwon *et al*., showed that glucose deprivation affected the reduced accumulation of HIF-1α under hypoxic conditions by disrupting translational processes in prostatic cancer cells^43^. In addition, Hubbi *et al*., also showed that chaperone-mediated autophagy promotes HIF-1α degradation after HCC cells are exposed to low glucose culture medium under hypoxic conditions^44^. Clinical metastasis prediction in tumor patients often fails when using a single parameter. For instance, Hoskin *et al*., failed to predict local control or metastasis-free survival by hypoxia markers in bladder cancer^45^.

To investigate the oxygen concentration-dependent changes in cellular proliferation and EMT, HepG2 cells were grown under different concentrations of oxygen (0%, 1%, 3%, and 21%) in our study. While HIF1α and GLUT1 were upregulated at less than 3% oxygen, the expression of PCNA, as a marker of proliferation, increased at 3% oxygen and started to reduce at 0% and 1% oxygen. EMT-related proteins began to be upregulated from below 3%. This suggested that cellular proliferation could be decreased in spite of increased GLUT1 expression. Using pimonidazole as a hypoxic marker, the xenograft model using HepG2 cells demonstrated that pimonidazole-stained hypoxic tumor regions showing high GLUT1 but low Ki67 expression had high EMT expression. Therefore, the hypoxia-induced decreased cellular proliferation was correlated with increased EMT-related protein expression. Gastric cancer patients with regional lymph node metastasis, distant metastasis, or lymphatic invasion were found to have significantly lower proliferation^46^. Moreover, upregulation of Twist1, an EMT activator, has been found to be associated with reduced skin cancer cell proliferation *in vivo*^47^. The reduction in cancer cell proliferation associated with the activation process of EMT is generally associated with increased resistance to chemotherapy that targets highly proliferating cells^24,48^.

There has been an emerging interest in the effects of metabolic processes on metastasis. The increased expression of GLUT1 upon exposure to hypoxia leads to a stimulation of glucose transport^49–51^. However, our *in vitro* and animal experiments showed that GLUT1 was consistently expressed in necrotic areas where glucose is no longer required for the production of cellular energy. A weak correlation was observed between hypoxic volume and hypermetabolic volume in non-small cell lung cancer patients^52^. Moreover, GLUT1 was expressed in tumor regions where EMT-related proteins were not increased. Thus, GLUT1 expression was necessary but not sufficient for induction of EMT process. Indeed, patients with HCCs with increased 18F-FDG uptake were correlated with increased GLUT1 expression. However, in line with the *in vitro/in vivo* data, HCCs with low Ki67 in spite of increased GLUT1 expression were correlated with increased EMT-related proteins, such as N-cadherin. These results might have an important implication in the use of 18F-FDG-PET/CT as a useful diagnostic tool for evaluating metastasis^49,53,54^. A previous study by our group found that increased 18F-FDG uptake in patients with HCC was associated with an increased frequency of metastasis^33^. However, only 10 out of 34 patients (29.4%) with increased 18F-FDG uptake in the primary tumor presented metastasis, while 69.6% did not. These results could be attributable to 18F-FDG uptake being indicative of the expression of GLUT1 but not cell proliferation and hypoxia. Recently, Zhang *et al*. reported that it is unknown whether 18F-FDG can be used to monitor liver metastasis of colorectal cancer^55^. Thus, to enhance the prediction of metastasis, parameters such as glucose and cell proliferation, have to be considered based on our results. Radiopharmaceuticals have investigated tumor biology. 18F-FMISO, which selectively binds to hypoxic cells in human tumors, has been used for staging and follow-up of multiple malignant tumors including glioblastoma, lung cancer, head and neck cancer, and breast cancer^56–59^. Currently, few tracers such as 18F-FLT measure proliferation. Saga *et al*. assessed the clinical potential of FLT-PET/CT imaging to detect cell proliferation in response to radiotherapy in patients with non-small cell lung carcinoma^60^. As we have proved above, one parameter cannot predict metastasis. Thus, based on our *in vitro* and *in vivo* experiments, we propose that combining 18F-FLT and FMISO with 18F-FDG PET can possibly provide an improved diagnostic and prognostic tool for metastasis and response to anti-cancer agents.

HCC is a heterogeneous disease, from both a clinical and a molecular viewpoint. In this study, we performed *in vitro* and *in vivo* analysis to determine the microenvironments that induce EMT process in hypoxic areas of liver cancer tissues. Contrary to other studies that attempted to link EMT process to only one condition, for example, oxygen concentration, the current study investigated integrated conditions that induce EMT process. Overall, our data revealed that HCC cells begin to induce EMT process or cell invasion when they encounter a combination of hypoxia, glucose deficiency, and reduced proliferation signals. These findings could be very useful in clinical practice during PET analysis for predicting the prognosis of metastasis and therapy outcome.

## Methods

### Human HCC samples

This study was approved by the Institutional Review Board at Yonsei University Health System Severance Hospital (Seoul, South Korea). All the experiments were performed following relevant guidelines and regulations (Yonsei IRB number: 4-2015-0904). Prior informed consents were obtained from all patients. We selected 23 curative surgical resection-treated patients in whom 18F-FDG PET/CT was performed for preoperative staging at our institution. The 18F-FDG uptake for the quantitative evaluation of HCC was performed as previously reported^33^. Among the 23 selected patients, 13 showed low 18F-FDG uptake (TLR, ≤1.62) and 10 showed high 18F-FDG uptake (TLR, >1.62).

### Cell culture and treatment

All cell lines were purchased from the Korean Cell Line Bank (Seoul, South Korea). HepG2 cells were cultured in Roswell Park Memorial Institute (RPMI) 1640 supplemented with 10% (v/v) foetal bovine serum (FBS) and 100 U penicillin/100 µg-streptomycin at 37 °C and 5% CO_2_ atmosphere. Hep3B and Huh7 cells were cultured in Dulbecco’s modified Eagle’s medium supplemented with 10% (v/v) FBS and 100 U penicillin/100 µg streptomycin at 37 °C and 5% CO_2_ atmosphere. All cells were routinely tested for mycoplasma and found to be contamination-free. Moreover, they were maintained in culture for a maximum of 5 passages after thawing. Hypoxia was achieved by incubating the cells in an incubator (STEMCELL Technologies Inc.) in which the oxygen was replaced by pure nitrogen. The gas proportions used were 21% O_2_: 21% O_2_ and 5% CO_2_; 3% O_2_: 3% O_2_, 5% CO_2_, and 92% N_2_; 1% O_2_: 1% O_2_, 5% CO_2_, and 94% N_2_ and 0% O_2_: 0% O_2_, 5% CO_2_, and 95% N_2_. Cells were cultured for indicated incubation time and were harvested for further analysis. The proliferation of HepG2 cells was estimated using a cell counting kit (CCK-8) from Dojindo Laboratories (Kumamoto, Japan). The HepG2 cells were cultured with various concentrations of glucose. After 24 h of incubation, HepG2 cells were incubated under hypoxic (1% oxygen) condition for additional 8 h. The absorbance was measured at a wavelength of 450 nm using a microplate reader (Molecular Devices, California, US). For small interfering RNA (siRNA)-mediated knockdown of PCNA, HepG2 cells were transfected with scrambled siRNA and siPCNA (Bioneer, Daejeon, Korea) using Lipofectamine following the manufacturer’s instruction. The sequences of siRNA targeting human PCNA are as follows: (i) sense: 5′- CAGCAUAUACUGAAGUCUUtt-3′; antisense: 5′- AAGACUUCAHUAUAUGCUGtt-3′.

### Invasion assay

The cell invasion assays were performed as described previously^33^. Invasion assays were performed using Corning® BioCoat™ Matrigel® Invasion Chamber (BioCoat; BD Biosciences, Heidelberg, Germany). A total of 100,000 cells in medium containing 0.1 mM or 25 mM glucose were added into the upper chamber of the insert with 8-mm pores in 24-well tissue culture plates. The lower well was filled with a medium supplemented with 25 mM glucose, and after 8 h, the invaded cells were fixed for 10 min (4% v/v formaldehyde in PBS) before staining with 0.1% crystal violet for 15 min, followed by washing with PBS. Images were acquired using an Olympus BX53 microscope with Olympus Cell Sens software (Carl Zeiss Microscopy, GmbH, Jena, Germany).

### Animal preparation

All animal procedures were performed in accordance with the guidelines of the Animal Care Committee at the Yonsei University Health System Institutional Animal Care and were approved by the Animal Care Committee at the Yonsei University Health System Institutional Animal Care prior to experiments (IACUC number 2015‐0195). Male BALB/c-nu mice of SPF grade, 4‒5-weeks old, were purchased from Central lab. Animal inc (Korea). HCC cells growing exponentially *in vitro* were trypsinised and harvested for tumor implantation. All animal manipulations were performed under sterile conditions. HCC cells (at a concentration of 100,000 cells/ml) at the logarithmic growth phase were washed twice with serum-free culture solution and suspended in Matrigel (BD Biosciences). The cells (0.1 ml) were inoculated subcutaneously on the back of each nude mouse. The animals were provided with sterilized food and water. The mice were weighed, and the tumor growth in nude mice was monitored carefully once a week. After 6 weeks, mice were injected intravenously (tail vein) with 60 mg/kg of pimonidazole solution (Hypoxyprobe™, Hypoxyprobe, Inc.). After 90 min, mice were sacrificed under anesthesia and dissected. Tumor tissues were collected and fixed using 4% paraformaldehyde for pathology study.

### Immunostaining

Immunohistochemistry (IHC) and immunofluorescence (IF) were performed as described previously^61^ using various antibodies (Supplementary Fig. S1). For immunocytochemistry (ICC), HepG2 cells were seeded on glass-bottomed cell culture dishes (NEST Biotechnology, Jiangsu, China) and incubated for 24 h under hypoxic conditions. Then, cells were fixed in 4% paraformaldehyde for 30 min and washed three times with PBS. Next, the cells were incubated in PBS containing 0.25% Triton X-100 for 10 min and blocked with blocking solution (3% bovine serum albumin, 0.25% Triton X-100 in PBS). Cells were treated for 30 min followed by incubation overnight at 4 °C with a diluted primary antibody against GLUT1 in blocking solution. The next day, the cells were washed three times with PBS and incubated with diluted secondary antibody (1:200, Invitrogen, Oregon, USA) in blocking solution for 1 h. After washing the cells in PBS, Hoechst 33258 was used to stain the nuclei. Images were acquired using an Olympus BX53 microscope with Olympus Cell Sens software (Carl Zeiss Microscopy, GmbH, Jena, Germany). Quantification of fluorescence in microscopic images stained with GFP (Green) and DsRed (Red) was carried out using IMT i-Solution software (Martin Microscope Company, Easley, USA).

### *In vitro* 18F-FDG uptake assay

HepG2 cells were plated into 6-well plates (150,000 cells/well). The following day, cells were incubated under hypoxic conditions. After 0, 1, 2, and 3 days, the culture medium was exchanged with glucose-free DMEM (Thermo Fisher Scientific). Approximately, 0.0037 MBq of 18F-FDG was added to the cells, which were then incubated for 20 min. The cells were then washed three times with PBS, after which sodium dodecyl sulfate (SDS) lysis buffer (60 mM Tris-HCl, pH 6.8, 1% SDS) was added to each well. The cell lysates were collected, and the amount of radioactivity incorporated into the cells was measured using a gamma counter (Perkin Elmer, Waltham, MA, USA). The measured radioactivity was normalized to the protein content in the cell lysates, which was calculated using the Nanodrop ND-1000 spectrophotometer (Denovix, Wilmington, USA).

### Protein extraction and western blotting

Total proteins were extracted from cells, and western blotting was performed as previously reported^33^. The primary antibodies used were: PCNA (Cell Signaling Technology, Danvers MA, USA; #13110; dilution 1:5000); Hexokinase II (EPR20839; 1:500), HIF1α(1:1000), GLUT1 (1:2000), N cadherin (5D5; 1:1000), and Snail/Slug (1:1000), all procured from Abcam (Cambridge, UK); and β-actin-HRP (Santa Cruz Biotechnology, Dalla TX, USA; C4; dilution 1:1000). Western blotting experiments from biological replicates showed similar expression data, attesting to the reproducibility of the results. For band quantification, images were analyzed using Image Lab software (Bio-Rad, Hercules, California, USA). For band quantification, images were analyzed using IMT i-Solution software Martin Microscope Company, Easley, USA).

### Real-time PCR

Total RNA was extracted with TRIzol (Invitrogen). cDNAs were synthesized from 500 ng of total RNA using the ReverTra Ace® qPCR RT Master Mix with gDNA Remover (TOYOBO, Osaka, Japan). Quantitative RT-PCR was conducted on C1000™ Thermal Cycler (Bio-Rad) using SYBR® Green Realtime PCR Master Mix (TOYOBO, Japan). The gene expression levels were normalized by beta-2 microglobulin (B2M) mRNA expression levels of corresponding cDNA samples. All PCR primers were purchased from Bioneer (Daejeon, Korea). The following primers were used: HIF1 (Forward 5′-CTGACCCTGCACTCAATCAAG-3′, Reverse 5′-TGGGACTATTAGGCTCAGGTG-3′), CDH2 (Forward 5′-GAGACTTGCGAAACTCCAGAC-3′, Reverse 5′-CATTAAGCCGAGTGATGGTCC-3′), PCNA (Forward 5′-TGTGCAAAAGACGGAGTGAAA-3′, Reverse 5′-AGTTCAGGTACCTCAGTGCAA-3′), SNAl1 (Forward 5′-CCCCAATCGGAAGCCTAACTA-3′, Reverse 5′-ACAGAGTCCCAGATGAGCATT -3′), B2M (Forward 5′-TTACTCACGTCATCCAGCAGA-3′, Reverse 5′-AGAAAGACCAGTCCTTGCTGA-3′).

### TCGA data analysis

mRNA levels were obtained from the Cancer Genome Atlas (TCGA) Liver hepatocellular carcinoma dataset available at the OncoLnc (www.oncolnc.org) TCGA data portal. A set of 360 HCC samples with high and low gene expression groups (50-50 percentile) was used for constructing graphs showing correlations between different genes. GraphPad Prism 5 (GraphPad Software, San Diego, CA, USA) was used for mapping.

### Statistical analysis

Statistical analyses were performed using GraphPad Prism Software (GraphPad Software, Inc., San Diego, CA). Results are expressed as mean ± SE (range). P values <0.05 were considered statistically significant. Comparisons between groups were made using the Mann-Whitney test.

## Supplementary information

Supplementary information.

## Data Availability

The datasets generated and/or analyzed during the current study are available from the corresponding author on reasonable request.

## References

[1] Fattovich G, Stroffolini T, Zagni I, Donato F (2004). Hepatocellular carcinoma in cirrhosis: incidence and risk factors. Gastroenterology.

[2] Ferlay J (2015). Cancer incidence and mortality worldwide: sources, methods and major patterns in GLOBOCAN 2012. Int. J. Cancer.

[3] Lu LC, Hsu CH, Hsu C, Cheng AL (2016). Tumor Heterogeneity in Hepatocellular Carcinoma: Facing the Challenges. Liver Cancer.

[4] Schulze K, Nault JC, Villanueva A (2016). Genetic profiling of hepatocellular carcinoma using next-generation sequencing. J. Hepatol..

[5] Ferlay J (2010). Estimates of worldwide burden of cancer in 2008: GLOBOCAN 2008. Int. J. Cancer.

[6] Velazquez RF (2003). Prospective analysis of risk factors for hepatocellular carcinoma in patients with liver cirrhosis. Hepatology.

[7] Mueller-Klieser W (2000). Tumor biology and experimental therapeutics. Crit. Rev. Oncol. Hematol..

[8] Sutherland RM (1988). Cell and environment interactions in tumor microregions: the multicell spheroid model. Sci..

[9] Totoki Y (2014). Trans-ancestry mutational landscape of hepatocellular carcinoma genomes. Nat. Genet..

[10] Schulze K (2015). Exome sequencing of hepatocellular carcinomas identifies new mutational signatures and potential therapeutic targets. Nat. Genet..

[11] Kan Z (2013). Whole-genome sequencing identifies recurrent mutations in hepatocellular carcinoma. Genome Res..

[12] Huang J (2012). Exome sequencing of hepatitis B virus-associated hepatocellular carcinoma. Nat. Genet..

[13] Guichard C (2012). Integrated analysis of somatic mutations and focal copy-number changes identifies key genes and pathways in hepatocellular carcinoma. Nat. Genet..

[14] Cleary SP (2013). Identification of driver genes in hepatocellular carcinoma by exome sequencing. Hepatology.

[15] Ahn SM (2014). Genomic portrait of resectable hepatocellular carcinomas: implications of RB1 and FGF19 aberrations for patient stratification. Hepatology.

[16] Lau CK (2009). An Akt/hypoxia-inducible factor-1alpha/platelet-derived growth factor-BB autocrine loop mediates hypoxia-induced chemoresistance in liver cancer cells and tumorigenic hepatic progenitor cells. Clin. Cancer Res..

[17] Daskalow K (2010). Role of hypoxia-inducible transcription factor 1alpha for progression and chemosensitivity of murine hepatocellular carcinoma. J. Mol. Med..

[18] Zhang L (2013). Hypoxia induces epithelial-mesenchymal transition via activation of SNAI1 by hypoxia-inducible factor -1alpha in hepatocellular carcinoma. BMC Cancer.

[19] Yang MH (2008). Direct regulation of TWIST by HIF-1alpha promotes metastasis. Nat. Cell Biol..

[20] Krishnamachary B (2006). Hypoxia-inducible factor-1-dependent repression of E-cadherin in von Hippel-Lindau tumor suppressor-null renal cell carcinoma mediated by TCF3, ZFHX1A, and ZFHX1B. Cancer Res..

[21] Tavanez JP, Valcarcel J (2010). A splicing mastermind for EMT. EMBO J..

[22] Kalluri R, Neilson EG (2003). Epithelial-mesenchymal transition and its implications for fibrosis. J. Clin. Invest..

[23] Thiery JP (2002). Epithelial-mesenchymal transitions in tumour progression. Nat. Rev. Cancer.

[24] Vega S (2004). Snail blocks the cell cycle and confers resistance to cell death. Genes. Dev..

[25] Evdokimova V, Tognon C, Ng T, Sorensen PH (2009). Reduced proliferation and enhanced migration: two sides of the same coin? Molecular mechanisms of metastatic progression by YB-1. Cell Cycle.

[26] Scheel C, Weinberg RA (2012). Cancer stem cells and epithelial-mesenchymal transition: concepts and molecular links. Semin. Cancer Biol..

[27] Mani SA (2008). The epithelial-mesenchymal transition generates cells with properties of stem cells. Cell.

[28] Zeisberg M, Neilson EG (2009). Biomarkers for epithelial-mesenchymal transitions. J. Clin. Invest..

[29] Ye X (2015). Distinct EMT programs control normal mammary stem cells and tumour-initiating cells. Nat..

[30] Lawson DA (2015). Single-cell analysis reveals a stem-cell program in human metastatic breast cancer cells. Nat..

[31] Yoon KT (2007). Role of 18F-fluorodeoxyglucose positron emission tomography in detecting extrahepatic metastasis in pretreatment staging of hepatocellular carcinoma. Oncol..

[32] Sugiyama M (2004). 18F-FDG PET in the detection of extrahepatic metastases from hepatocellular carcinoma. J. Gastroenterol..

[33] Lee M, Jeon JY, Neugent ML, Kim JW, Yun M (2017). 18F-Fluorodeoxyglucose uptake on positron emission tomography/computed tomography is associated with metastasis and epithelial-mesenchymal transition in hepatocellular carcinoma. Clin. Exp. Metastasis.

[34] Jeon JY (2018). Regulation of Acetate Utilization by Monocarboxylate Transporter 1 (MCT1) in Hepatocellular Carcinoma (HCC). Oncol. Res..

[35] Kelman Z (1997). PCNA: structure, functions and interactions. Oncogene.

[36] Kelly BD (2003). Cell type-specific regulation of angiogenic growth factor gene expression and induction of angiogenesis in nonischemic tissue by a constitutively active form of hypoxia-inducible factor 1. Circ. Res..

[37] Lee M, Ko H, Yun MJ (2018). Cancer Metabolism as a Mechanism of Treatment Resistance and Potential Therapeutic Target in Hepatocellular Carcinoma. Yonsei Med. J..

[38] Zhang, L. *et al*. Hypoxia induces epithelial-mesenchymal transition via activation of SNAI1 by hypoxia-inducible factor-1 alpha in hepatocellular carcinoma. *Bmc Cancer* **13**, doi:Artn 10810.1186/1471-2407-13-108 (2013). 10.1186/1471-2407-13-108 PMC3614870 23496980

[39] Xiang ZL (2011). Gene Expression Profiling of Fixed Tissues Identified Hypoxia-Inducible Factor-1 alpha, VEGF, and Matrix Metalloproteinase-2 as Biomarkers of Lymph Node Metastasis in Hepatocellular Carcinoma. Clin. Cancer Res..

[40] Zheng, S. S., Chen, X. H., Yin, X. & Zhang, B. H. Prognostic Significance of HIF-1 alpha Expression in Hepatocellular Carcinoma: A Meta-Analysis. *Plos One* **8**, doi:ARTN e6575310.1371/journal.pone.0065753 (2013). 10.1371/journal.pone.0065753 PMC3683060 23799043

[41] Amann T (2008). GLUT1 expression is increased in hepatocellular carcinoma and promotes tumorigenesis. J. Hepatology.

[42] Sun, H. W. *et al*. GLUT1 and ASCT2 as Predictors for Prognosis of Hepatocellular Carcinoma. *Plos One* **11**, doi:ARTN e016890710.1371/journal.pone.0168907 (2016). 10.1371/journal.pone.0168907 PMC5201247 28036362

[43] Kwon SJ, Lee YJ (2005). Effect of low glutamine/glucose on hypoxia-induced elevation of hypoxia-inducible factor-1alpha in human pancreatic cancer MiaPaCa-2 and human prostatic cancer DU-145 cells. Clin. Cancer Res..

[44] Hubbi ME (2013). Chaperone-mediated autophagy targets hypoxia-inducible factor-1alpha (HIF-1alpha) for lysosomal degradation. J. Biol. Chem..

[45] Hoskin PJ, Sibtain A, Daley FM, Wilson GD (2003). GLUT1 and CAIX as intrinsic markers of hypoxia in bladder cancer: relationship with vascularity and proliferation as predictors of outcome of ARCON. Br. J. Cancer.

[46] Lee HE, Kim MA, Lee BL, Kim WH (2010). Low Ki-67 proliferation index is an indicator of poor prognosis in gastric cancer. J. Surg. Oncol..

[47] Tsai JH, Donaher JL, Murphy DA, Chau S, Yang J (2012). Spatiotemporal regulation of epithelial-mesenchymal transition is essential for squamous cell carcinoma metastasis. Cancer Cell.

[48] Mejlvang J (2007). Direct repression of cyclin D1 by SIP1 attenuates cell cycle progression in cells undergoing an epithelial mesenchymal transition. Mol. Biol. Cell.

[49] Ebert BL, Firth JD, Ratcliffe PJ (1995). Hypoxia and mitochondrial inhibitors regulate expression of glucose transporter-1 via distinct Cis-acting sequences. J. Biol. Chem..

[50] Murakami T (1992). Identification of two enhancer elements in the gene encoding the type 1 glucose transporter from the mouse which are responsive to serum, growth factor, and oncogenes. J. Biol. Chem..

[51] Dang CV, Semenza GL (1999). Oncogenic alterations of metabolism. Trends Biochem. Sci..

[52] Sachpekidis C (2015). Combined use of (18)F-FDG and (18)F-FMISO in unresectable non-small cell lung cancer patients planned for radiotherapy: a dynamic PET/CT study. Am. J. Nucl. Med. Mol. Imaging.

[53] Na SJ (2017). (18)F-FDG PET/CT Can Predict Survival of Advanced Hepatocellular Carcinoma Patients: A Multicenter Retrospective Cohort Study. J. Nucl. Med..

[54] Lee JW (2016). Prognostic Significance of (1)(8)F-FDG Uptake in Hepatocellular Carcinoma Treated with Transarterial Chemoembolization or Concurrent Chemoradiotherapy: A Multicenter Retrospective Cohort Study. J. Nucl. Med..

[55] Zhang M (2018). Noninvasive evaluation of (18)F-FDG/(18)F-FMISO-based Micro PET in monitoring hepatic metastasis of colorectal cancer. Sci. Rep..

[56] Spence AM (2008). Regional hypoxia in glioblastoma multiforme quantified with [18F]fluoromisonidazole positron emission tomography before radiotherapy: correlation with time to progression and survival. Clin. Cancer Res..

[57] Norikane T (2014). Correlation of (18)F-fluoromisonidazole PET findings with HIF-1alpha and p53 expressions in head and neck cancer: comparison with (18)F-FDG PET. Nucl. Med. Commun..

[58] Cheng J (2013). 18F-fluoromisonidazole PET/CT: a potential tool for predicting primary endocrine therapy resistance in breast cancer. J. Nucl. Med..

[59] Arvold ND, Heidari P, Kunawudhi A, Sequist LV, Mahmood U (2016). Tumor Hypoxia Response After Targeted Therapy in EGFR-Mutant Non-Small Cell Lung Cancer: Proof of Concept for FMISO-PET. Technol. Cancer Res. Treat..

[60] Saga T (2011). PET/CT with 3′-deoxy-3′-[18F]fluorothymidine for lung cancer patients receiving carbon-ion radiotherapy. Nucl. Med. Commun..

[61] Lee M (2013). Transcriptome analysis of MENX-associated rat pituitary adenomas identifies novel molecular mechanisms involved in the pathogenesis of human pituitary gonadotroph adenomas. Acta Neuropathol..

