# Supplementary material for: The critical role of glucose deprivation in epithelial-mesenchymal transition in hepatocellular carcinoma under hypoxia
Source: Sci Rep. 2020 Jan 30;10:1538. doi: 10.1038/s41598-020-58124-1 (PMC6992695; doi:10.1038/s41598-020-58124-1)

**The critical role of glucose deprivation in epithelial-mesenchymal transition in hepatocellular carcinoma under hypoxia**

Hanhee Jo^1,#^, Jongsook Lee^1,#^, Jeongyong Jeon^2^, Seon yoo Kim^2^, Jee-in Chung^2^, Hae yong Ko^2^, Misu Lee^1,^*, Mijin Yun^2,^*

^1^Division of Life Sciences, College of Life Science and Bioengineering, Incheon National University, Incheon, South Korea

^2^Department of Nuclear Medicine, Severance Hospital, Yonsei University College of Medicine, Seoul, South Korea

^#^These two authors contributed equally to this work.

**Keywords:** hepatocellular carcinoma, metastasis, hypoxia, glucose deficiency

*: To whom correspondence should be addressed.

Misu Lee, PhD

Incheon National University, Incheon, Korea

E-mail: misulee@inu.ac.kr

Tel.: +82 32 835 8091

Fax: + 8232 835 0754

Mijin Yun, MD, PhD

Yonsei University College of Medicine, Seoul, 120-749, Korea

E-mail: yunmijin@yuhs.ac

Tel.: +82 2 2123 5851

Fax: + 82 2 365 5882

**Supplementary Figure Legends**

**Supplementary Fig S1. List of antibodies**

**Supplementary Fig S2. Quantification of fluorescence using microscopic images.** We quantified the positive fluorescence indicating the expression of Hif1α (A), GLUT1 (B), and CD31 (C) from 23 patients with high FDG uptake (n=10) and low FDG uptake (n=13) in Fig 1A and 1B. Quantification of fluorescence in microscopic images stained with GFP (Green) and DsRed (Red) was carried out using IMT i-Solution software (Martin Microscope Company, Easley, USA). (D) We counted the Ki67 positive cells in positive and negative tumor regions of indicated proteins in HCC with high FDG uptake. Data expressed as the mean ± SD.;**, P < 0.01.

**Supplementary Fig S3. The expression of CAIX, GLUT1 and Ki67 in** in HCC xenograft models. (A) The expression of CAIX, Ki67, and GLUT1 in HCC xenograft models. Immunohistochemistry using FFPE tissues was performed using the indicated antibodies. Nuclei were counterstained with hematoxylin. Scale bars: 100 μm. (B, C) Expression of CAIX and GLUT1 (B) and of Ki67 and GLUT1 in HCC xenograft model. Immunostaining with antibodies against indicated proteins performed on FFPE tissues. Nuclei were counterstained with DAPI. Scale bars: 50μm

**Supplementary Fig S4. The correlation of indicated mRNAs of patients with HCC.** (A) RNA was extracted from frozen HCC samples obtained after trans-sphenoidal surgery. RT-PCR was performed using probe sets specific for *HIF1α*, *PCNA*, *SNAI1*, and *CDH2*. The expression levels of the target genes were normalized to that of *B2M* (housekeeping gene) and fold change was calculated using the 2^−ΔΔCt^ method. (B) The indicated mRNA levels were obtained from the Cancer Genome Atlas (TCGA) Liver Hepatocellular Carcinoma dataset available on the OncoLnc (www.oncolnc.org) TCGA data portal.

**Supplementary Fig. S5. Reduction of cell proliferation after glucose deprivation under 1% hypoxic condition.** HepG2 cells were incubated with various concentrations of glucose under hypoxia (1% oxygen). After 8 h, cell proliferation was measured by CCK-8 assays. Data are shown as the mean of three independent experiments ± SD.

**Supplementary Fig. S6.** Increased EMT-related protein expression after glucose-deprivation under hypoxia. (A) Hep3B cells were incubated with various concentrations of glucose under normoxia or hypoxia (1% oxygen). After 8 h, the HIF1α, GLUT1, HK2, N-cadherin, Snail/Slug, PCNA, and β-actin expression levels were examined by western blotting. The grouped images have been cropped from images of different blots exposed for the same time. (B) HepG2 cells were transfected with MOCK or pCMV3- Hif1α. After 24 h, HepG2 cells were incubated with the indicated concentration of glucose under hypoxia (1% oxygen) for 8 h. The HIF1α, N-cadherin, Snail/Slug, and β-actin expression levels were then examined by western blotting. The grouped images have been cropped from those of different blots exposed for the same time. (C) Hep3B cells were incubated with the indicated concentration of glucose under hypoxia (1% oxygen) for 8 h. Hep3B cells penetrating the membrane were fixed and visualized by the staining of crystal violet. (D) Quantitative analyses were performed for the cells migrating through the matrigel-coated filter. Five random fields of each test at × 200 magnifications were counted (±standard deviation). **p < 0.01

**Supplementary Fig S7. Hif1α expression after treatment with glutamine.** (A) HepG2 cells were incubated with indicated condition of medium under hypoxia. After 8 hours, the HIF1α and β-actin expression levels were then examined by western blotting. (B) Immunohistochemistry with indicated antibodies performed on FFPE tissues from HepG2-xenograft model. Scale bars: 50μm.

**Supplementary Fig. S8.** Increased EMT-related protein expression in low proliferative cells under hypoxia. (A) Hep3B cells were transfected with scrambled siRNA oligos or siRNA oligos against PCNA. After 24 h, HepG2 cells were incubated with various concentrations of glucose under hypoxia (1% oxygen) for 8 h. The PCNA, HIF1α, N-cadherin, Snail/Slug, and α-actin expression levels were then examined by western blotting.

**Supplementary Fig S1.**

| **Antibodies** | **Company** | **Cat. no.** | **Clonality** | **Clone no.** | **Dilution** |
| --- | --- | --- | --- | --- | --- |
| CA9 | GeneTex | GTX70020 | Mouse monoclonal | GT12 | 1:500 |
| CD31 | abcam | ab9498 | Mouse monoclonal | JC/70A | 1:100 |
| GLUT1 | abcam | ab652 | Rabbit polyclonal |  | 1:500 |
| HIF1α | abcam | ab2185 | Rabbit polyclonal |  | 1:500 |
| Ki67 | Dako | M7240 | Mouse monoclonal | MIB-1 | 1:500 |
| N-cad | abcam | ab98952 | Mouse monoclonal | 5D5 | 1:200 |
| E-cad | Santacruz | sc-21791 | Mouse monoclonal |  | 1:500 |
| Pimonidazol | hpi | HP FITC MAb-1 | Mouse monoclonal | 4.3.11.3 | 1:200 |
| Vimentin | santacruz | sc-373717 | Mouse monoclonal | E-5 | 1:500 |

**Supplementary Fig S2.**

**
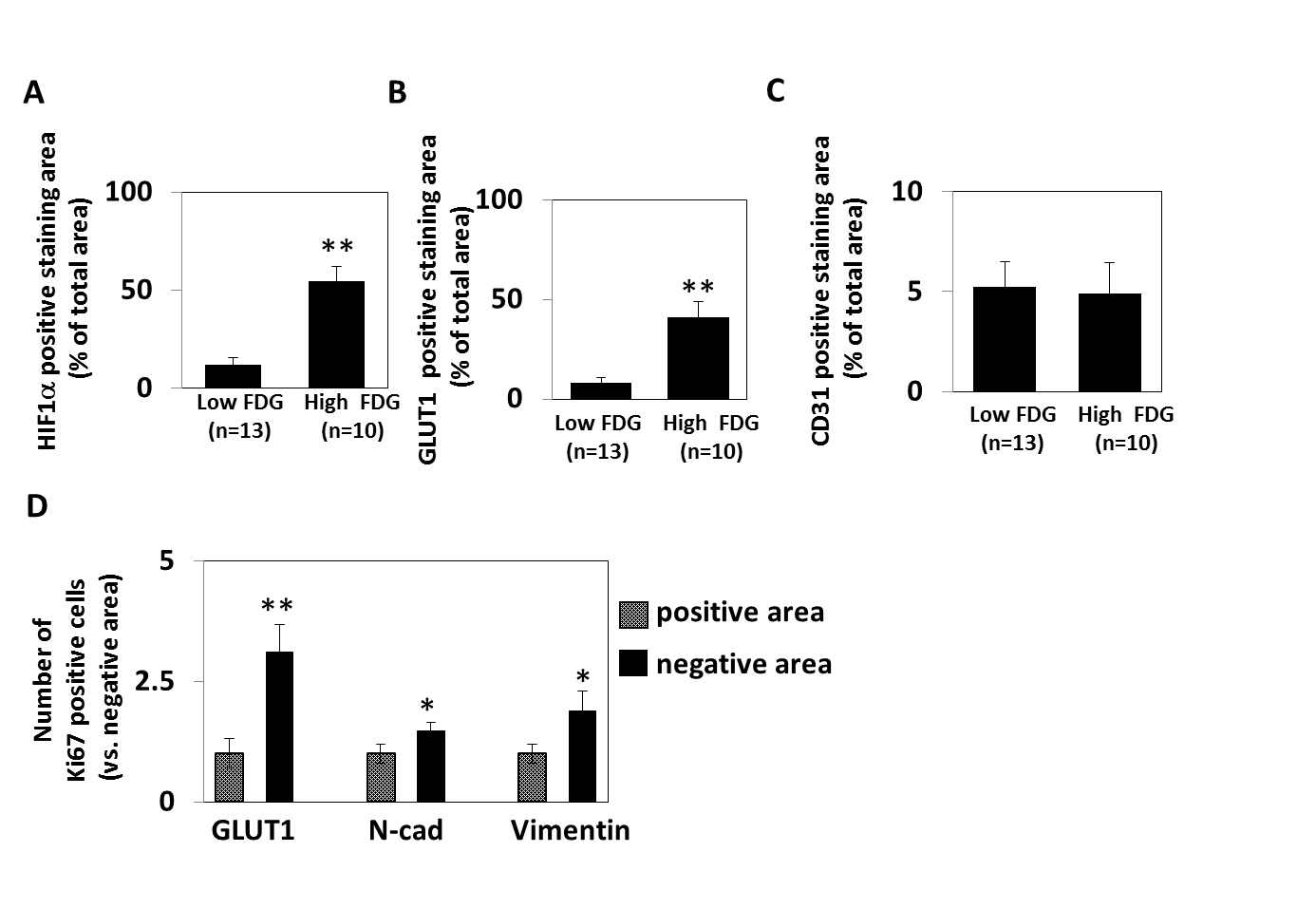
**

**Supplementary Fig S3.**

**
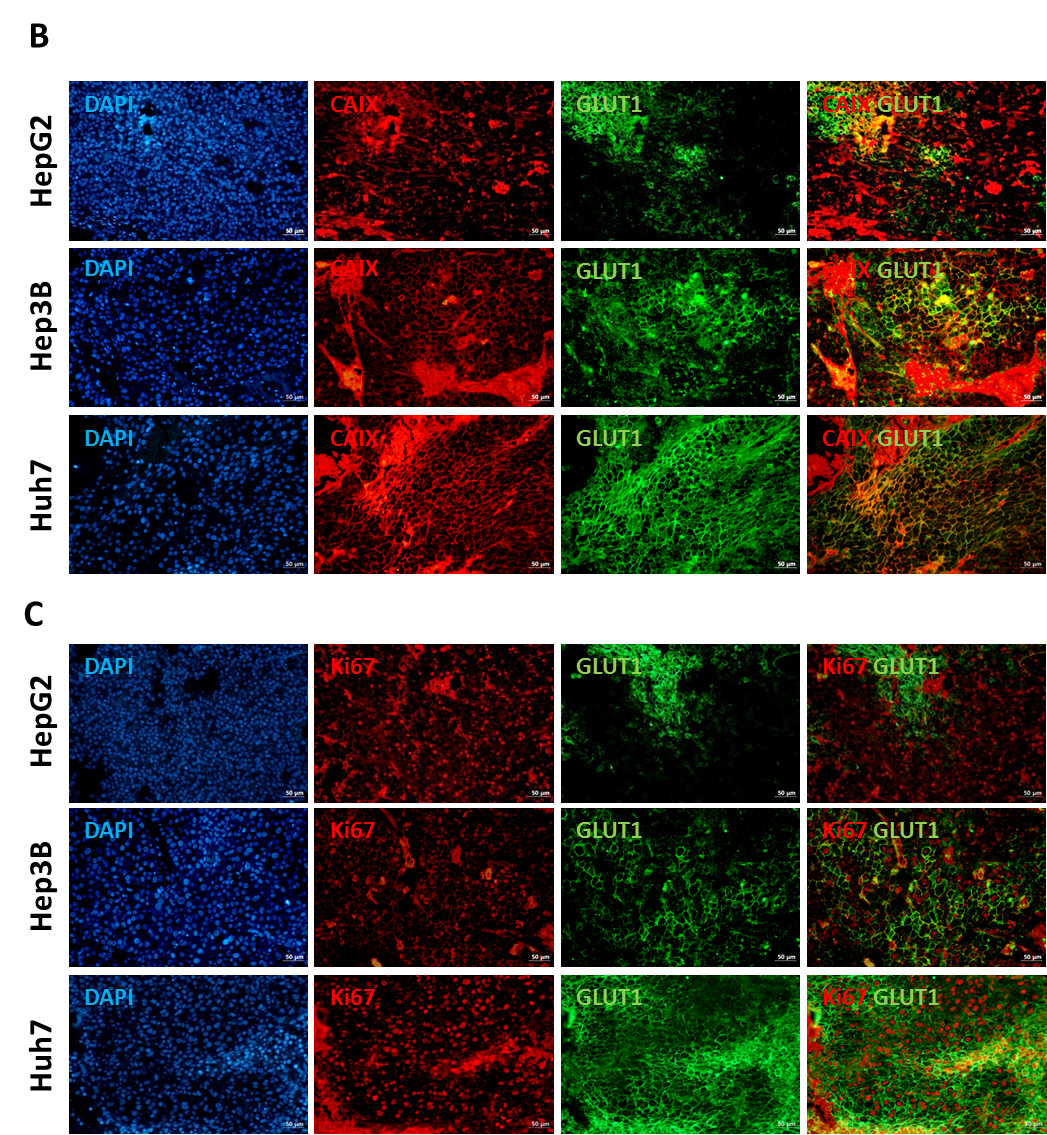

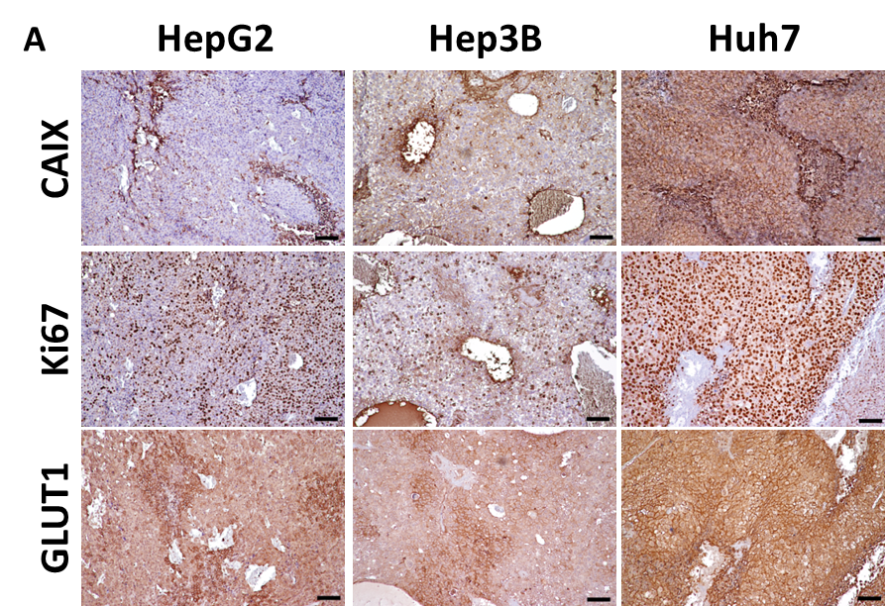
**

**Supplementary Fig S4.**

**A**

**
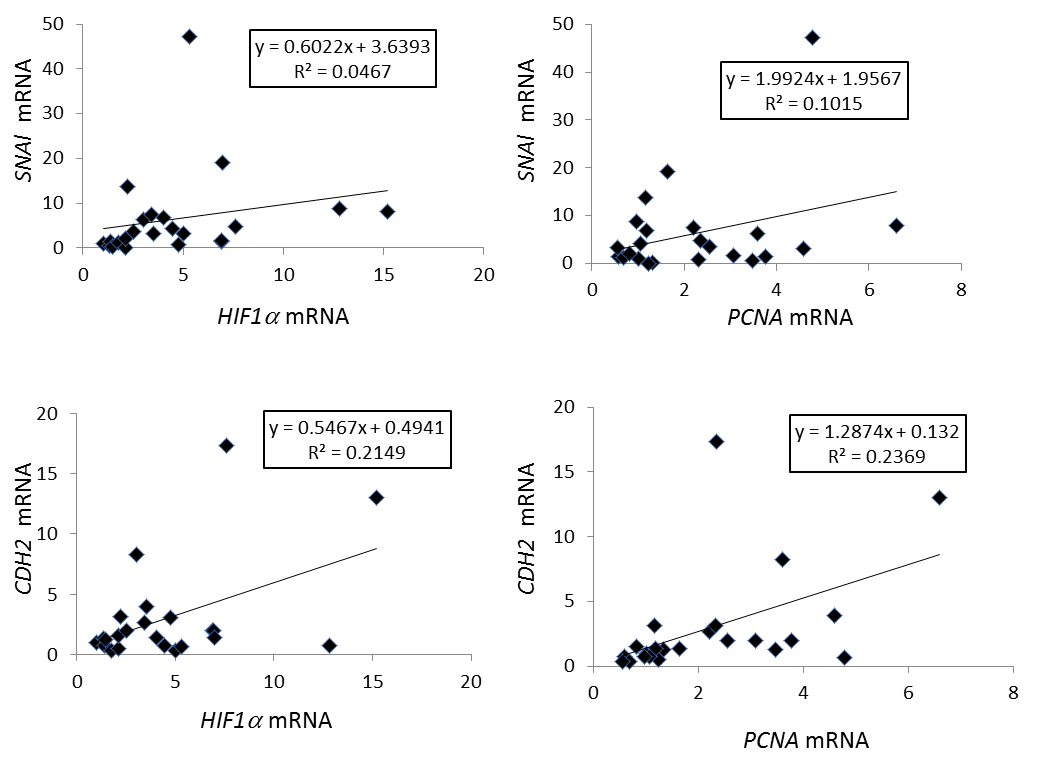
**

**
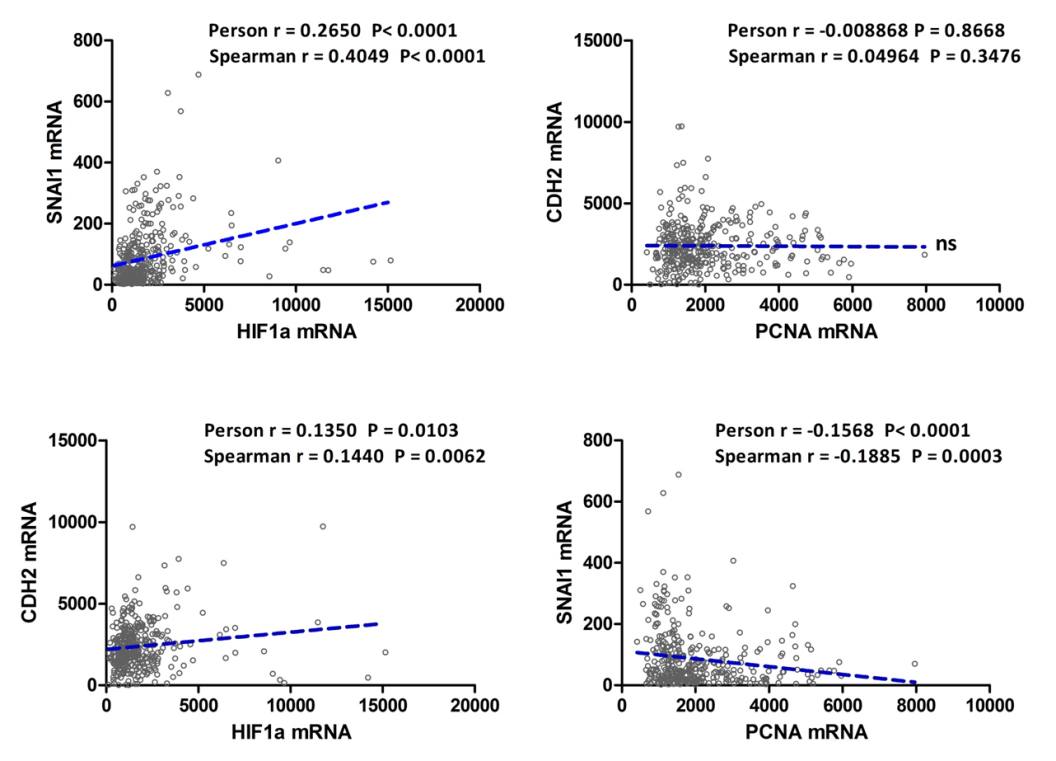
**

**B**

**Supplementary Fig S5.**

**
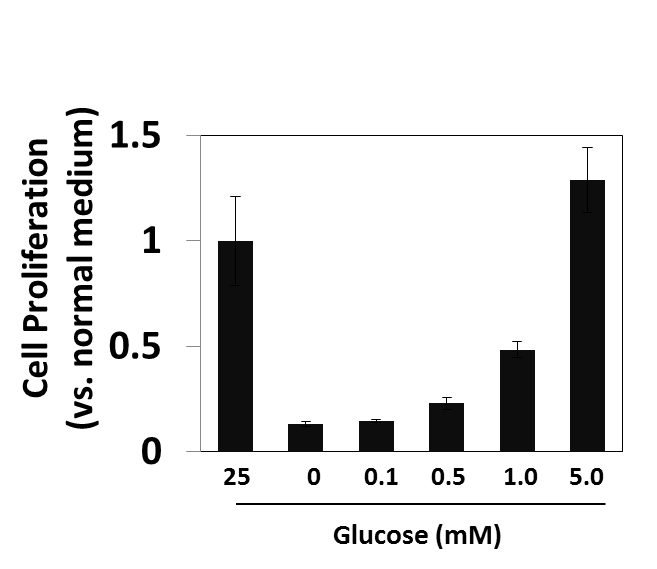
**

**Supplementary Fig S6.**

**
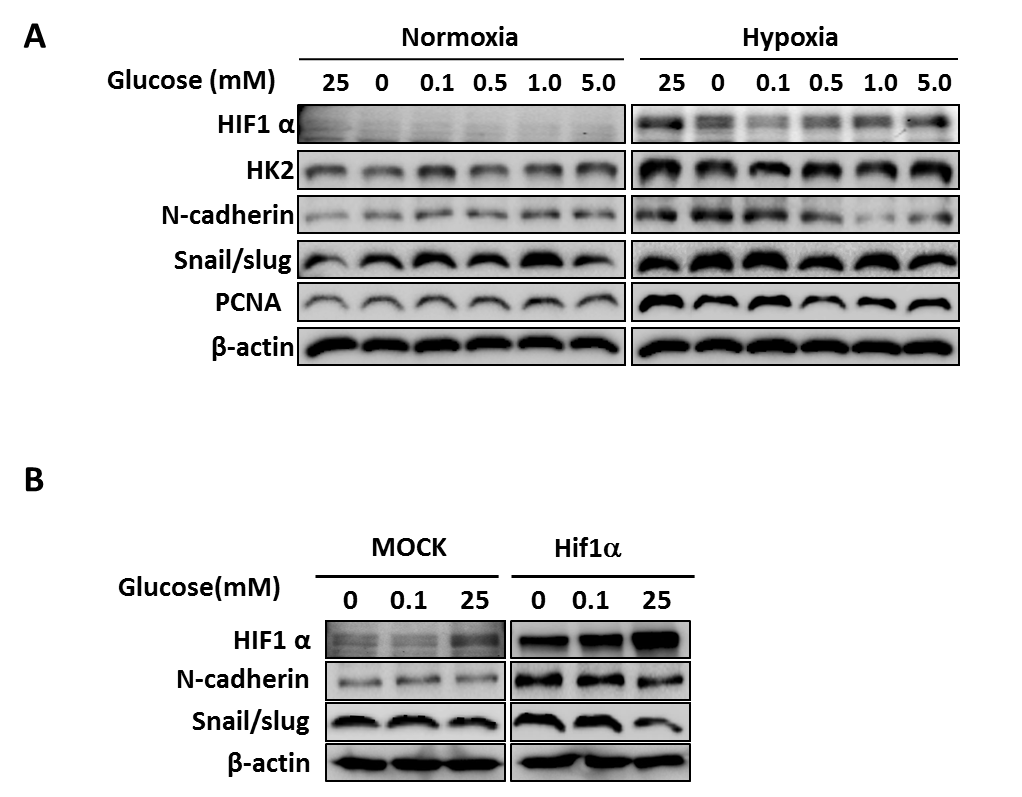
**

**
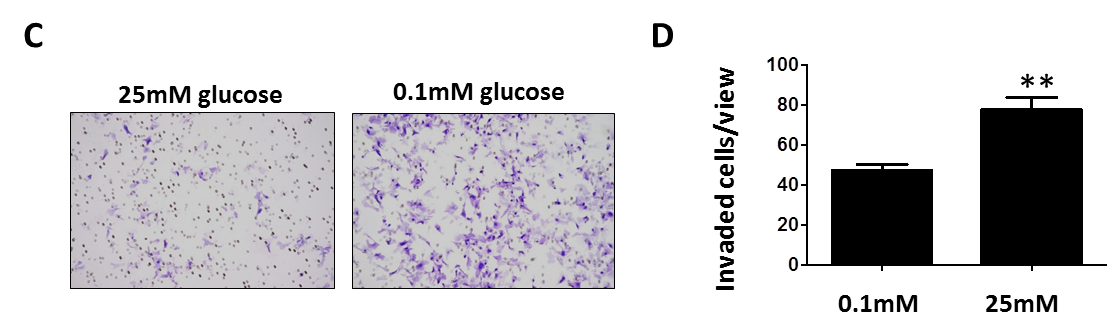
**

**Supplementary Fig S7.**

**A**


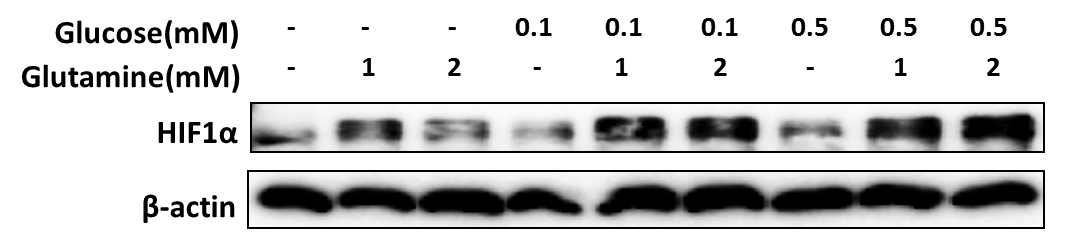


**
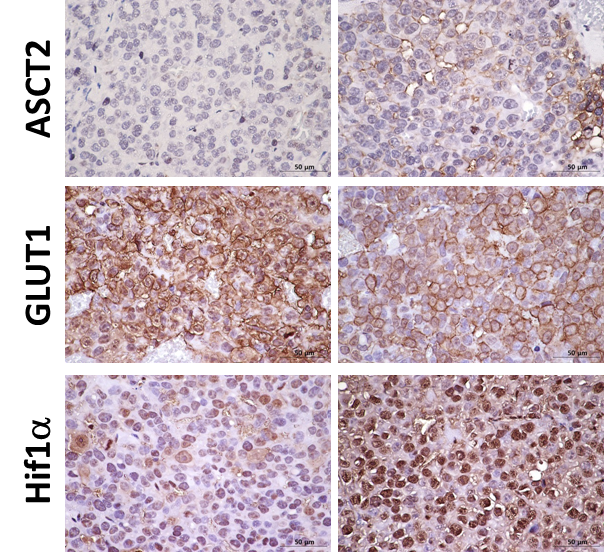
B**

**Supplementary Fig S8.**

**A**


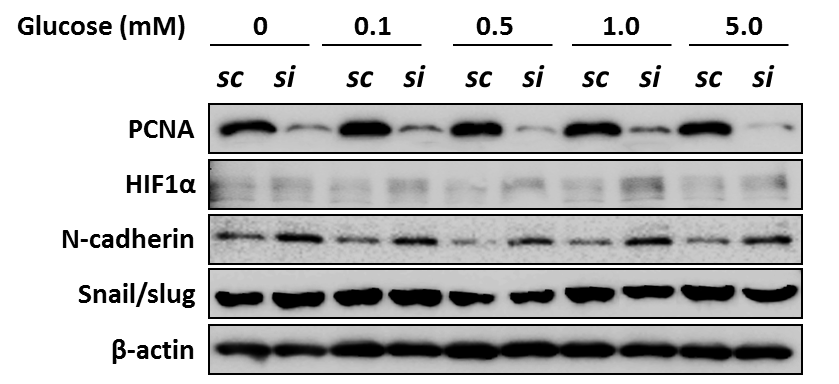

Supplement: Supplementary file 1 — Supplementary information. [file 41598_2020_58124_MOESM1_ESM.docx]
